# Fungal Pacemaker Endocarditis Caused by Candida albicans: An Unusual Case

**DOI:** 10.7759/cureus.97787

**Published:** 2025-11-25

**Authors:** Nidia Oliveira, Sofia Camões, Elisa Veigas, Tânia Batista, Catarina Oliveira

**Affiliations:** 1 Internal Medicine, Unidade Local de Saúde de Viseu Dão-Lafões, Viseu, PRT

**Keywords:** antifungals, candida albicans, candida endocarditis, fungal endocarditis treatment, pacemaker lead, surgical explantation

## Abstract

Fungal pacemaker endocarditis (FPE) is a rare but potentially fatal infection that can occur in patients with implanted cardiac devices. Diagnosing FPE can be difficult due to its subtle and non-specific symptoms, which often necessitate heightened clinical awareness to identify. Management typically involves removal of the infected device combined with antifungal therapy.

We describe a 79-year-old female patient who developed *Candida albicans* endocarditis involving a pacemaker lead, successfully managed through coordinated surgical extraction of the device and systemic antifungal therapy.

## Introduction

Infective endocarditis (IE) primarily involves the cardiac valves and endocardial surface, but can also affect intracardiac prosthetic materials such as implanted electronic devices [1]. It is predominantly associated with host predisposing conditions (such as diabetes mellitus or immunosuppression) or the presence of risk factors, including prosthetic heart valves, cardiac implantable electronic devices, previous cardiac surgeries, catheter-related infections, prolonged fungemia, or extended use of broad-spectrum intravenous antibiotics [2-4]. It carries a substantial risk of morbidity and mortality, especially when appropriate treatment is delayed [1,2]. Bacteria are the predominant causative agents of IE, with Staphylococcus and Streptococcus species accounting for nearly 80% of all reported cases [2]. Fungal pathogens may also be involved in the etiology of IE, representing 2% to 4% of all reported cases [1,2,5].

Fungal endocarditis (FE) comprises a small proportion of IE cases but is linked to a mortality rate over 70% [6,7]. Among fungal etiologies, Candida species predominate, responsible for approximately 80% of cases, while Aspergillus species are less frequently isolated [4,5]. Vegetations are typically large in size, posing a high risk of peripheral embolization, a complication reported in up to 44% of cases, and which constitutes the leading cause of death in these patients [8].

Clinically, this form of endocarditis often presents in a subacute manner, with prolonged fever (lasting more than two weeks) of unknown etiology. It may occur in isolation or be accompanied by constitutional symptoms such as general malaise, sweating, and fatigue, which are frequently indistinguishable from those seen in bacterial endocarditis. This overlap requires a high degree of clinical suspicion for the diagnosis to be considered [3-5]. The diagnostic process is often challenging, as blood cultures may be negative or show delayed fungal growth [9-11]. Transesophageal echocardiography is the imaging modality of choice for detecting vegetations associated with pacemaker leads [9,11].

Lead-related endocarditis may develop through direct inoculation during device placement or via bloodstream dissemination from a distant source. These mechanisms facilitate pathogen adherence to the leads and subsequent biofilm formation [3]. It is also considered a rare condition, with a reported incidence ranging from 0.13% to 19.9% in the literature [12].

The treatment of FE requires an aggressive therapeutic approach, ideally initiated early, combining prolonged antifungal therapy with surgical removal of the infected device [12].

We report the case of a 79-year-old woman diagnosed with FE due to *Candida albicans*, characterized by a large mass adherent to a pacing lead in the right ventricle. The combination of systemic antifungal therapy and surgical explanation of the pacing system led to a favorable clinical outcome.

This case highlights the importance of maintaining a high degree of suspicion for FE in patients with cardiac implantable electronic devices, emphasizing that early diagnosis and prompt surgical management are crucial for improving clinical outcomes.

## Case presentation

A 79-year-old woman with a history of invasive adenocarcinoma of the gastric antrum, arterial hypertension, dyslipidemia, type 2 diabetes mellitus, and a permanent pacemaker implanted in 2021 due to second-degree atrioventricular block.

She had previously undergone a partial gastrectomy on September 30, 2024, with a 76-day hospitalization during which she developed multiple complications, including acute kidney injury KDIGO2, nosocomial pneumonia, electrolyte disturbances (hypernatremia), and, most notably, a *C. albicans* fungemia, likely originating from a central venous catheter. She was treated with fluconazole for 15 days, with clinical resolution, and was discharged.

On February 26, 2025, approximately two months after discharge, the patient was readmitted with a seven-day history of high-grade fever (>39.5 °C), myalgia, and generalized malaise. On initial evaluation, her vital signs were blood pressure 98/60 mmHg, heart rate 80 bpm, temperature 36 °C, and oxygen saturation 99% on room air. She denied respiratory, urinary, gastrointestinal, or other symptoms suggestive of an alternative infectious source.

Laboratory tests showed markedly elevated inflammatory markers: leukocytosis (14.9 × 10⁹/L), neutrophilia (93%), procalcitonin 21.9 ng/mL, and C-reactive protein 7.4 mg/dL. Urinalysis and chest X-ray were unremarkable.

**Figure 1:**
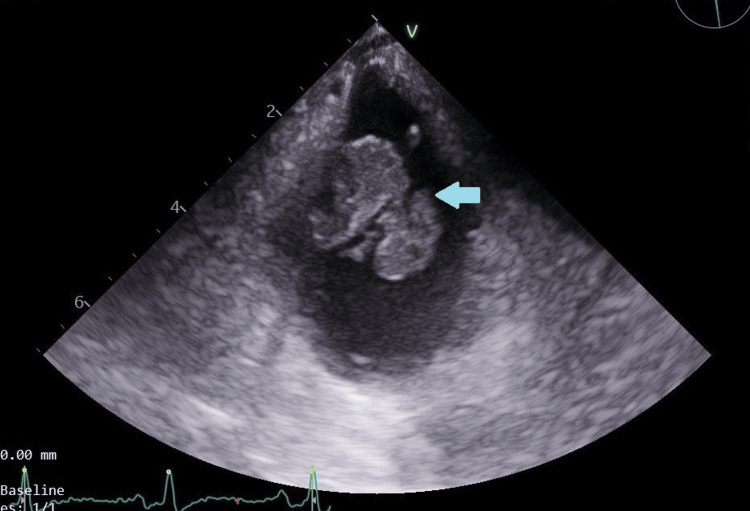
Transesophageal echocardiogram showing a large vegetation (arrow) measuring 23 × 19 mm, attached to the pacemaker lead in the right ventricle.

New blood cultures were collected, and fluconazole therapy was initiated based on the previous isolation of *C*. *albicans* from blood cultures during the prior hospitalization. The patient was admitted to the Internal Medicine Department for treatment and further evaluation. On the second day of hospitalization, blood cultures revealed yeast growth, later identified as *C. albicans*. A transesophageal echocardiogram was performed, revealing a large, complex, and friable bilobed mass (measuring 23 × 19 mm) adherent to the ventricular pacing lead, suggestive of a vegetation (Figure 1).

Given the severity of the condition, antifungal therapy was escalated to amphotericin B (3 mg/kg/day) combined with flucytosine (25 mg/kg every six hours), which she continued for six weeks. Cardiology was consulted due to the urgent need for surgical intervention, and on April 2, 2025, she underwent cardiac surgery, including replacement of the pacemaker and leads. Subsequent blood cultures were negative, and a follow-up transthoracic echocardiogram showed no vegetations or complications related to the previous device-associated endocarditis.

The patient demonstrated progressive clinical improvement, with no significant symptoms, and was discharged with follow-up planned in the Cardiology outpatient clinic.

## Discussion

The increasing use of cardiac implantable electronic devices (CIEDs), such as pacemakers and defibrillators, has led to a rise in device-associated infections [8,12]. Although CIED-related endocarditis remains relatively uncommon (accounting for approximately 10% of cases), it is a serious and potentially life-threatening condition [4]. The most common pathogens are Staphylococcus species (responsible for 60-80% of cases); however, fungal infections, particularly those caused by *Candida albicans*, though rare, are associated with high morbidity and mortality [4]. *C. albicans* is notable for its ability to form dense biofilms on surfaces such as cardiac devices, which complicates treatment due to antifungal resistance and the difficulty of eradicating the infection with medical therapy alone [3].

FE seldom occurs in immunocompetent hosts. Predisposing factors include diabetes mellitus, renal impairment, immunosuppressive therapy, intravenous drug use or prolonged antibiotic exposure, prosthetic valves or prior cardiac surgery, and recent hospitalization [12].

In the case presented, the patient’s main predisposing factors included diabetes mellitus, the presence of a pacemaker, and a recent hospitalization for subtotal gastrectomy, which was complicated by *Candida albicans* fungemia, most likely originating from a central venous catheter.

Patients generally exhibit nonspecific symptoms, with sustained fever being predominant. Peripheral markers of endocarditis, like Osler nodes or Janeway lesions, are seldom observed [3]. The high embolic potential of fungal vegetations is related to their large and fragile structure [4].

Echocardiography is essential for the diagnosis of IE, with transesophageal echocardiography being the preferred modality for detecting vegetations on intracardiac devices due to its superior sensitivity compared to transthoracic echocardiography [12]. Like the case from Karrati et al., our patient also had a large vegetation (23 × 19 mm) adherent to a pacemaker lead, suggestive of a vegetation, but our case additionally demonstrates a clear link to a prior catheter infection. Although blood cultures are crucial for both diagnosis and treatment, their sensitivity may be limited in FE, with no organism isolated in over 50% of cases [3,4].

The treatment of Candida endocarditis requires an aggressive, combined therapeutic approach. Current recommendations of the European Society of Cardiology (ESC), the American Heart Association (AHA) and the Infectious Diseases Society of America (IDSA) advise treatment with liposomal amphotericin B, either alone or combined with flucytosine, or alternatively with high-dose echinocandins for a minimum duration of six weeks [3]. Transition to oral fluconazole may be appropriate in patients infected with fluconazole-susceptible strains who demonstrate clinical improvement and clearance of Candida from the bloodstream. Surgical removal of the infected device is essential for therapeutic success and relapse prevention [3].

In the present case, management followed these recommendations, with removal of the pacemaker and administration of liposomal amphotericin B and flucytosine for six weeks, resulting in favorable clinical and laboratory outcomes.

## Conclusions

Although rare, FE carries a high risk of embolic events and is associated with significant morbidity and mortality, requiring early diagnosis and aggressive management. A combination of transesophageal echocardiography, serial blood cultures, and, in some cases, analysis of surgical specimens is essential for diagnostic confirmation.

In patients with pacemakers or other CIEDs who present with fever or bacteremia, the possibility of endocarditis should always be considered. This case highlights the importance of early recognition of FE in patients with CIEDs and associated risk factors, as well as the need for prompt multidisciplinary intervention to improve clinical outcomes in a condition that remains challenging to treat.
